# Structural Basis for the Limited Response to Oxidative and Thiol-Conjugating Agents by Triosephosphate Isomerase From the Photosynthetic Bacteria *Synechocystis*

**DOI:** 10.3389/fmolb.2018.00103

**Published:** 2018-11-27

**Authors:** Eduardo Castro-Torres, Pedro Jimenez-Sandoval, Eli Fernández-de Gortari, Margarita López-Castillo, Noe Baruch-Torres, Marisol López-Hidalgo, Antolín Peralta-Castro, Corina Díaz-Quezada, Rogerio R. Sotelo-Mundo, Claudia G. Benitez-Cardoza, L. Michel Espinoza-Fonseca, Adrian Ochoa-Leyva, Luis G. Brieba

**Affiliations:** ^1^Laboratorio Nacional de Genómica para la Biodiversidad, Centro de Investigación y de Estudios Avanzados del IPN, Guanajuato, Mexico; ^2^Division of Cardiovascular Medicine, Department of Internal Medicine, Center for Arrhythmia Research, University of Michigan, Ann Arbor, MI, United States; ^3^Laboratorio de Investigación Bioquímica, Programa Institucional en Biomedicina Molecular ENMyH-IPN, Ciudad de México, Mexico; ^4^Laboratorio de Estructura Biomolecular, Centro de Investigación en Alimentación y Desarrollo, A.C., Hermosillo, Mexico; ^5^Departamento de Microbiología Molecular, Instituto de Biotecnología, Universidad Nacional Autónoma de México, Cuernavaca, Mexico

**Keywords:** triosephosphate isomerase, X-ray structure, thiol-based redox regulation, oxidative damage, protein evolution

## Abstract

In plants, the ancestral cyanobacterial triosephosphate isomerase (TPI) was replaced by a duplicated version of the cytosolic TPI. This isoform acquired a transit peptide for chloroplast localization and functions in the Calvin-Benson cycle. To gain insight into the reasons for this gene replacement in plants, we characterized the TPI from the photosynthetic bacteria *Synechocystis* (SyTPI). SyTPI presents typical TPI enzyme kinetics profiles and assembles as a homodimer composed of two subunits that arrange in a (β-α)_8_ fold. We found that oxidizing agents diamide (DA) and H_2_O_2_, as well as thiol-conjugating agents such as oxidized glutathione (GSSG) and methyl methanethiosulfonate (MMTS), do not inhibit the catalytic activity of SyTPI at concentrations required to inactivate plastidic and cytosolic TPIs from the plant model *Arabidopsis thaliana* (AtpdTPI and AtcTPI, respectively). The crystal structure of SyTPI revealed that each monomer contains three cysteines, C47, C127, and C176; however only the thiol group of C176 is solvent exposed. While AtcTPI and AtpdTPI are redox-regulated by chemical modifications of their accessible and reactive cysteines, we found that C176 of SyTPI is not sensitive to redox modification *in vitro*. Our data let us postulate that SyTPI was replaced by a eukaryotic TPI, because the latter contains redox-sensitive cysteines that may be subject to post-translational modifications required for modulating TPI's enzymatic activity.

**Graphical Abstract:**
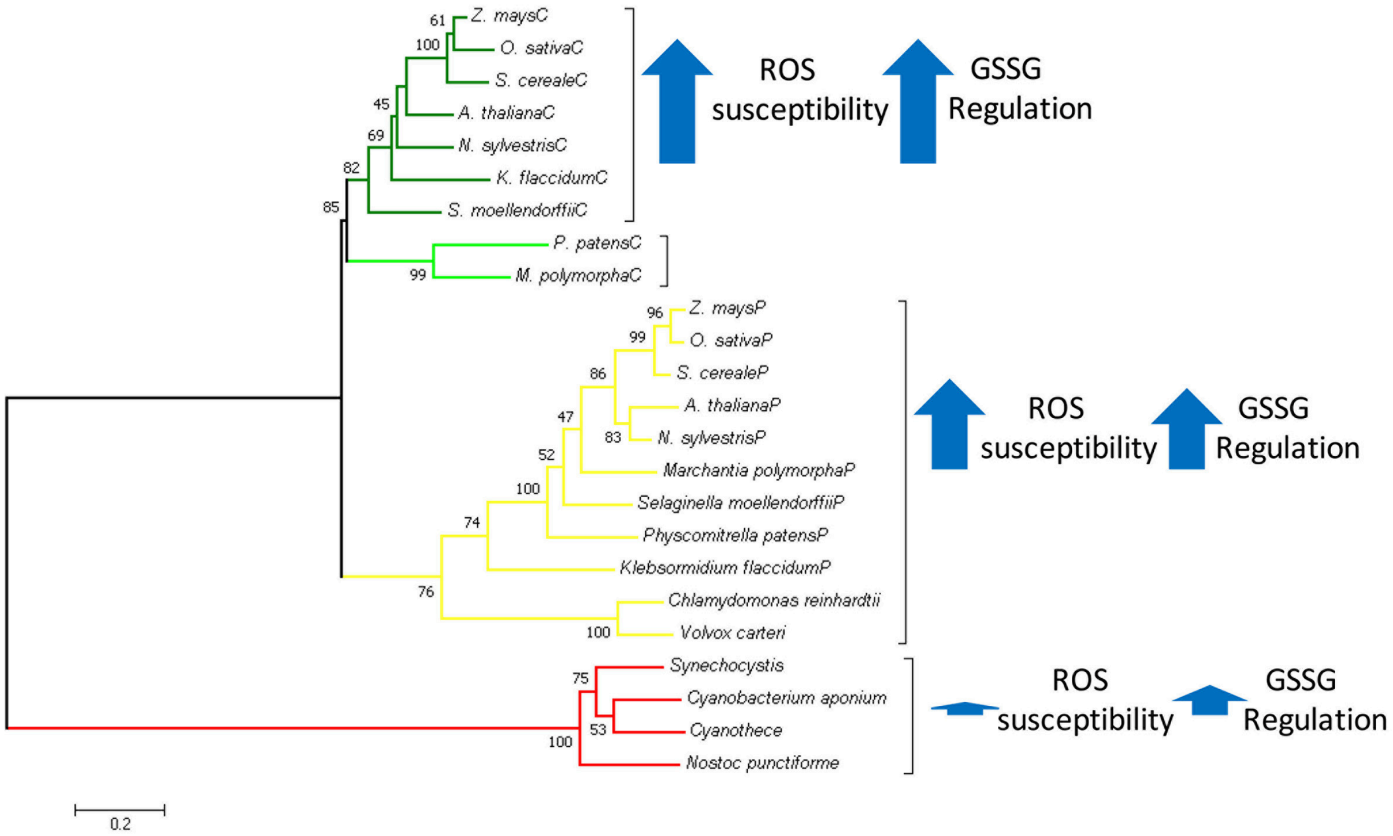
In Green Plantae chloroplastic triosephosphate isomerase evolved as a duplicated version of cytosolic TPI replacing the ancestral cyanobacterial enzyme. With basis on structural and biochemical data, we propose that this replacement was because the cyanobacterial TPI could not be regulated by redox-agents.

## Introduction

Cyanobacteria execute oxygenic photosynthesis and are believed to be the ancestors of chloroplast in algae and land plants (Gould et al., 2008). *Synechocystis* sp. strain PCC 6803 (*Synechocystis*) is able to growth phototrophically and heterotrophically and is the model system to study cyanobacteria (Kaneko et al., 1996). Cyanobacteria harbor three main metabolic pathways to degrade glucose: the oxidative pentose phosphate (OPP), the Embden–Meyerhof–Parnas (EMP) pathway or glycolysis, and the Entner–Doudoroff (ED) pathway (Chen et al., 2016). All pathways produce ATP, NADH, and building blocks for amino acids, and carbohydrate biosynthesis. In photosynthetic organisms, the OPP pathway runs in a reverse leading to the synthesis of carbohydrates in the Calvin–Benson cycle.

Triosephosphate isomerase (TPI) interconverts glyceraldehyde-3-phosphate (G3P) and dihydroxyacetone phosphate (DHAP) near diffusion rate-limiting during glycolysis and the Calvin-Benson cycle (Albery and Knowles, 1976; Blacklow et al., 1988). In most organisms, TPI is an obligated dimer that folds into a (β-α)_8_ barrel known as the TIM barrel (Walden et al., 2001; Wierenga et al., 2010). TPIs can be regulated by post-translational modifications, non-competitive regulation, and dimer-monomer equilibrium among other factors (Ito et al., 2003; Grüning et al., 2014; Zaffagnini et al., 2014; Lara-Gonzalez et al., 2015).

The cellular localization of TPIs in photosynthetic organisms is contrasting. In land plants, glycolysis and the Calvin-Benson cycle are compartmentalized and these organisms harbor two TPIs, one localized in the cytosol and one that translocates into the chloroplast. In green algae, the first steps of glycolysis, until the interconversion between G3P and DHAP, occur in the chloroplast and consequently, these organisms only harbor one TPI. In photosynthetic prokaryotes, both glycolysis and the Calvin-Benson cycle take place in the cytoplasm and these organisms also only need one TPI (Johnson and Alric, 2013).

It is estimated that nearly 1,000 genes derived from cyanobacteria were transferred into the nucleus of land plants and those genes are imported back as proteins into the chloroplast (Sato et al., 2005; Bock and Timmis, 2008; Gross et al., 2008). However, three essential genes for the Calvin-Benson cycle were replaced by their eukaryotic counterparts: TPI, fructose-1,6-bisphosphatase (FBP), and ribose 5-phosphate isomerase (RPI) (Reyes-Prieto and Bhattacharya, 2007). Thus, in land plants the ancestral cyanobacterial TPI originally present in chloroplasts was replaced by a duplicated version of the cytosolic TPI. The reasons for these replacements are unknown.

Post-translational modifications, such as reversible redox modifications, regulate protein activity during the Calvin-Benson cycle, both *in vitro* and *in vivo* (Zaffagnini et al., 2007; Michelet et al., 2008, 2013; Hall et al., 2010; Friso and Van Wijk, 2015; McConnell et al., 2018). However, non-reversible modifications in response to reactive oxygen species (ROS) and reactive nitrogen species may lead to loss of enzymatic function or protein aggregation (Dahl et al., 2015; Hao et al., 2016). The thiol group of cysteine is particularly reactive to hydrogen peroxide (H_2_O_2_), the hydroxyl radical (OH^·^), and the superoxide anion (${\text{O}}_{2}^{-}\cdot$). Cysteine oxidization could lead to irreversible modifications in the form of sulfinic and sulfonic acids. TPIs from the photosynthetic eukaryotes *Chlamydomonas reinhardtii* and *A. thaliana* have contrasting fates upon redox post-translational modifications. *C. reinhardtii* TPI (CrTPI) and the plastidic TPI isoform of *A. thaliana* (AtpdTPI) are resistant to oxidative agents, like H_2_O_2_ or diamide, whereas the cytosolic TPI isoform of *A. thaliana* (AtcTPI) is highly susceptible to redox modifications (Zaffagnini et al., 2014; Dumont et al., 2016; Lopez-Castillo et al., 2016). We hypothesize that these differences in susceptibility to redox agents explain the replacement of the cyanobacterial *tpi* gene by a duplicated version of the eukaryotic gene. Herein we found that *Synechocystis* TPI (SyTPI) is highly resistant to oxidative agents, suggesting that TPIs from prokaryotic photosynthetic organisms evolved to cope with ROS at the expense of redox regulation.

## Materials and methods

### *Synechocystis* TPI subcloning and protein purification

The nucleotide coding sequence of *Synechocystis* sp. PCC 6803 TPI (GenBank: BAK50928.1) was codon optimized and synthetically synthesized for its expression in *E. coli*. The gene product was subcloned into the *Nde* I and *Bam* HI restriction sites of a modified pET19 vector in which the enterokinase site is replaced by a PreScission Protease site cleavage sequence. Plasmids containing wild-type and point mutants of SyTPIs were used to transform an *E. coli* BL21 (DE3) Δ*tpi* strain (Sullivan et al., 2011) in Luria Bertani agar medium supplemented with 100 μg/ml of ampicillin. A single overnight colony was used to inoculate 100 ml of liquid Luria-Bertani (LB) medium supplemented with ampicillin and this overnight culture was used to inoculate 2 L of liquid LB. Cell cultures were grown until they reached an OD_600_ of 0.6 and induced with 1 mM isopropyl-β-D-thiogalactoside (IPTG). Bacteria cells were harvested by centrifugation and resuspended in 25 ml of lysis buffer [100 mM triethanolamine (TEA) pH 7.4, 0.1 M NaCl, 0.5 mM PMSF, and 0.1 mg ml−1 lysozyme]. The resuspended cell culture was incubated on ice for 20 min and then sonicated with 3 pulses of 30 s. Bacterial lysate was clarified by centrifugation at 24,400 g for 30 min at 4°C. The supernatant was passed through a 1 ml HisTrap column previously equilibrated with 100 mM TEA pH 7.4, 0.1 M NaCl. The column was washed with 20 column volumes and SyTPI was eluted with 2 ml of 100 mM TEA pH 7.4, 0.1 M NaCl, 500 mM imidazole. The eluate was dialyzed in 1 L of 0.1 M TEA pH 7.4, 50 mM NaCl, 2 mM dithiothreitol (DTT), 2 mM EDTA at 4°C with the addition of PreScission Protease. SyTPI was further purified by gel filtration in a Superdex 75 10/300 GL column (GE Healthcare Life Sciences) equilibrated with 0.1 M TEA pH 7.4, 50 mM NaCl, 2 mM DTT, 1 mM EDTA. Proteins were concentrated to 5 mg/ml and stored at 4°C for no more than 2 weeks. Previous to all biochemical assays, all TPIs were reduced with 20 mM DTT for 1 h at room temperature. Excess DTT was removed by using a prepacked Sephadex G-25 column equilibrated with 25 mM TEA pH 7.4, 150 mM NaCl.

### Enzyme kinetics

SyTPI concentration was measured using a molar extinction coefficient of 23,950 M−1 cm−1. Briefly, the catalytic activity of SyTPI and its mutants was measured spectrophotometrically by a coupled enzyme assay assisted by α-glycerophosphate dehydrogenase (α-GDH) in the direction of glyceraldehyde 3-phosphate (G3P) to dihydroxyacetone phosphate (DHAP) (Rozacky et al., 1971). Assays were performed in 0.1 TEA pH 7.4 and 10 mM EDTA with the addition of 0.2 mM NADH (nicotinamide adenine dinucleotide), 1 μg α-GDH, and 1 mM of D-L glyceraldehyde 3-phosphate. The reaction was started by addition of SyTPIs and enzymatic activity was calculated by the decrease in absorbance at 340 nm. The determination of the kinetic constants, K_M_ and k_cat_, were carried varying G3P concentrations from 0 to 5 mM.

### Site-directed mutagenesis

Site-directed mutagenesis was performed using the Q5 Site-Directed Mutagenesis Kit (New England Biolabs) accordingly to the manufacturer's instructions using the oligonucleotides described in Table S1. Mutagenesis was confirmed by DNA sequencing.

### Protein crystallography and structural determination

Crystallization experiments were initiated with SyTPI concentrated to 15 mg ml-1 and incubated with 5 mM of 2-phosphoglyceric acid (2-PG) using the hanging drop method. Rod-shaped crystals appeared after 4 days in a reservoir solution containing 0.2 M calcium acetate hydrate, 0.1 M sodium cacodylate trihydrate pH 6.5, and 18% w/v polyethylene glycol 8,000. Crystals growth for 1 week until they reached an approximate size of 700 × 70 × 70 μm. Protein crystals were quickly dipped into a cryoprotectant solution containing 15% of glycerol and 85% of reservoir solution and immediately flash-cooled in liquid nitrogen. Diffraction was collected on a Micromax 002+ diffractometer (Rigaku) equipped with a sealed tube conventional X-ray source. A single dataset was integrated and scaled using XDS and XSCALE, respectively. Phases were solved by molecular replacement using the program PHASER (McCoy et al., 2007) and the crystal structure of TPI from *Geobacillus stearothermophilus* (PDB code: 1BTM) as the search model. Initial model and refinement were executed using COOT and PHENIX. Structural coordinates were deposited with PDB accession number 6BVE.

### Fluorescence-based thermal-shift assay (TSA)

TSA was performed on a real-time PCR device (Step One Instrument 48 wells, Applied Biosystems) (Huynh and Partch, 2015). Briefly, purified proteins were diluted in 25 mM Tris buffer pH 8.0 and 100 mM NaCl to a final concentration of 4 μM. Nine microliters of 4 μM protein solution were mixed with 1 μL of Sypro Orange dye 20X. Thus, the final concentration of Sypro Orange dye in the sample was 2X and final volume was 10 μL. Measurements were recorded using an excitation of 490 nm and fluorescence was recorded at 575 nm. The melting curve was set from 25 to 95°C, increasing the temperature by 1°C every 2 min and recording the fluorescence. Data was obtained subtracting the experimental intensities from a control with no added protein. Data were analyzed using the Protein Thermal Shift software from Applied Biosystems. Assays were performed in triplicate.

### 2-nitro-5-thiocyanobenzoic acid (NTCB) cysteine footprinting

NTCB cleavage was performed as previously described (Jacobson et al., 1973). Purified proteins were incubated for 1 h at 37°C in reduction buffer (50 mM Tris-HCl pH 8.0, 100 mM NaCl, and 5 mM DTT). Reduced proteins were dialyzed against 50 mM Tris-HCl pH 8.0 and 100 mM NaCl to remove excess DTT. A 10-fold molar excess of NTCB over molar concentration of total cysteines was added to each sample. After incubation of 2 h at 20°C proteins were dialyzed against sample buffer to remove non-reacted NTCB. For denaturation, proteins were buffer exchanged into unfolding buffer (50 mM Tris-HCl pH 8.0, 100 mM NaCl, and 5 M urea). Cleavage was initiated with a raising of the pH of the reaction to pH 9.0 with 1 M NaOH. The reactions were incubated overnight at 37°C, stopped by addition of 3 mM β-mercaptoethanol and analyzed by SDS-PAGE.

### Western blot for SyTPI glutathionylation

Reduced wild-type and SyTPI mutants were concentrated to 6 μM and incubated in 100 mM TEA pH 7.4, 10 mM EDTA, and 1 mM glutathione-disulfide (GSSG) 48 h at 4°C. Excess of GSSG was removed by passing the treated proteins throughout a prepacked Sephadex G-25 column equilibrated with the incubation buffer without GSSG. Protein samples were separated by non-reducing SDS-PAGE gel, transferred to a nitrocellulose membrane and analyzed by Western blot using a primary mouse anti glutathione monoclonal antibody (1:250, Virogen) and a secondary antibody anti-mouse coupled to peroxidase (1:5,000, Thermo scientific). Signals were analyzed by chemiluminescence with CL-Xposure Film (Thermo Scientific, USA).

### Spectrophotometric determination of reactive thiols

The number of free thiols of SyTPI was determined spectrophotometrically with 5, 5′-Dithiobis (2-nitrobenzoic acid) (DTNB) (Ellman, 1959). For this assay, 90 μL of previously quantified protein was added to a solution containing 200 μM of DTNB (SIGMA, USA) in 100 mM Na_2_PO_4_ pH 8.0. The absorbance at 412 nm was determined after 5 min. of incubation at room temperature. An L-cysteine calibration curve was used to calculate the concentration of titrated sulfhydryl groups.

### Effect of redox agents on SyTPI activity

Reduced proteins were diluted to 184 nM in 0.1 M TEA pH 7.4, 10 mM EDTA and increasing concentrations of GSSG (0–25 mM), S-methyl methanethiosulfonate (MMTS) (0–50 μM), H_2_0_2_ (0–500 μM), and diamide (DA) (0–1,000 μM). The samples were incubated for 4 h at 20°C. After incubation, proteins were diluted 1,000-fold to a final concentration of 0.184 nM (10 ng/ml) and the activity was measured at 340 nm.

### Ornidazole inhibition

SyTPI and HsTPI at a concentration of 0.184 nM were incubated with increased ornidazole concentration from 0 to 50 μM. The samples were incubated for 60 min at room temperature and substrate was added to the reaction mixture. A continuous lecture was taken from 0 to 30 min. Substrate consumption after 5 min (to assure linearity) was taken as representative point. The inhibitory effect of ornidazole using SyTPI and HsTPI with mutations in the predicted ornidazole binding site of SyTPI were tested at 50 μM of ornidazole. Wild-type and mutants TPIs were incubated with 50 μM of ornidazole for 1 h at room temperature. We measured the activity in the presence and absence of ornidazole. The calculated activity corresponds to the ratio of both activities.

### Phylogenetic analysis

Representative amino acid sequences of TPIs from photosynthetic organism were used to run a multiple sequence alignment using the program MUSCLE (Edgar, 2004). The phylogenetic analysis was performed with the maximum likelihood method using 1,000 bootstrap replicates, all of the bioinformatics analysis were conducted on MEGA (Kumar et al., 2016).

### GdnHCl induced denaturation

Protein solutions were prepared at varying GdnHCl concentrations, ranking from 0 to 6.0 M and using 0.1 M increments. The samples were incubated at 25°C for 24 h (when equilibrium was reached). Afterwards, intrinsic fluorescence emission spectra were obtained using a LS-55 Spectrofluorometer (Perkin-Elmer), equipped with a water-jacketed cell holder for temperature control. All the experiments were performed using cells with a path-length of 1.0 cm, at 25°C. The excitation wavelengths were 280 or 295 nm (as indicated) and the emission spectra were collected from 320 to 400 nm. The fluorescence spectral center of mass (SCM) was calculated from the fluorescence intensity data (I_λ_), obtained at different wavelengths (λ) from 320 to 400 nm, using the expression: SCM = Σ (λ ^*^ I_λ_)/Σ I_λ_ (Sánchez-Miguel et al., 2010). Denaturation profiles were analyzed according to a two-state transition folding model for a dimeric protein, where denaturation and dissociation of native protein (N2) to form unfolded monomers (D) occur simultaneously as previously reported (Lara-González et al., 2014).

### Ensemble docking simulations

We used the ensemble docking approach to identify a consensus ornidazole-binding site on SyTPI and HsTPI. We first performed molecular dynamics (MD) simulations of both SyTPI and HsTPI in solution to explicitly model the flexibility of the enzyme required for the ensemble docking simulations. We used the crystal structures of SyTPI (this study) and HsTPI (PDB code: 1HTI) as starting structures for the MD simulations. We performed independent 100-ns MD simulations of apo SyTPI and HsTPI in a solution containing ~0.1 mM NaCl. MD simulations were performed with the program NAMD 2.12 (Phillips et al., 2005) and the CHARMM36 force field topologies and parameters (Best et al., 2012). For the ensemble docking simulations, we extracted a total of 85 and 101 individual structures from the MD trajectories of SyTPI and HsTPI, respectively. We first performed blind docking calculations with Autodock Vina (Trott and Olson, 2010) to search for ornidazole-binding sites on the entire surface of both SyTPI and HsTPI. The ornidazole-binding sites with the best docking scores were further selected for a new round of focused docking simulations. For these focused docking calculations, we used 50-point grid maps for each ornidazole-binding site discovered through blind docking simulations, each one with a grid space < 0.4 Å.

## Results

### *Synechocystis* TPI is resistant to oxidative and thiol-conjugating agents

**Figure 1:**
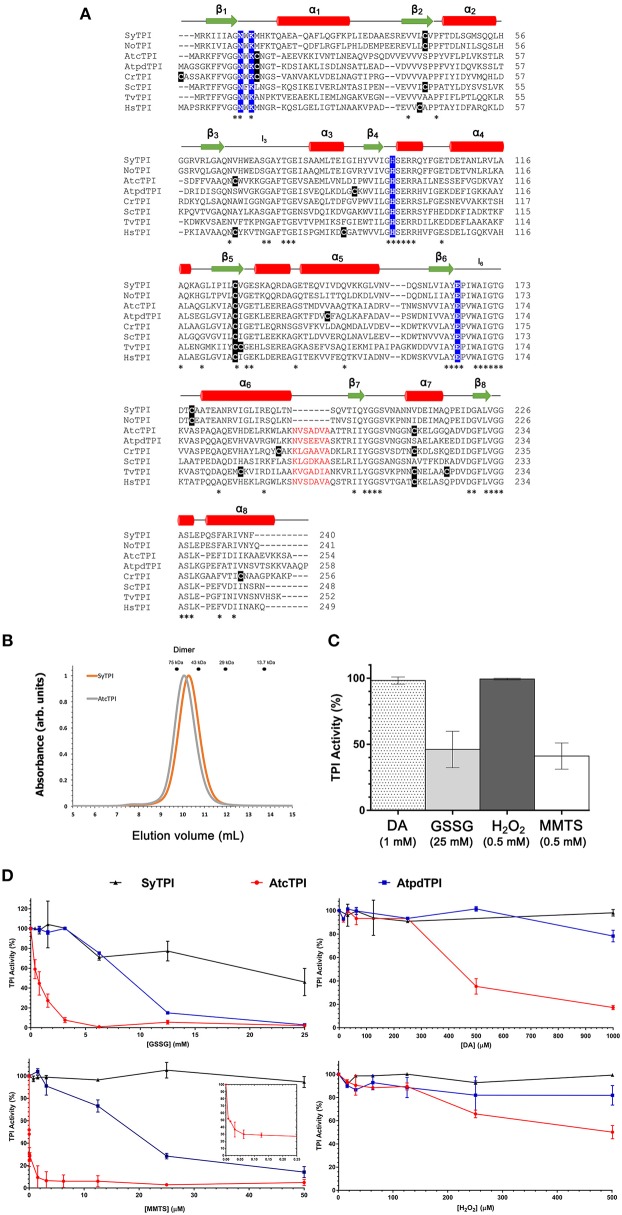
**(A)** Multiple sequence alignment and secondary structure elements of TPIs from diverse organisms: SyTPI (*Synechocystis* sp. PCC 6803), NoTPI, (*Nostoc* sp. PCC 7107), AtcTPI, (*Arabidopsis thaliana* cytosolic TPI), AtpdTPI (*Arabidopsis thaliana* chloroplastic TPI), CrTPI (*Chlamydomonas reinhardtii*), ScTPI (*Saccharomyces cerevisiae*), TvTPI (*Trichomonas vaginalis*), and HsTPI (*Homo sapiens*). Conserved residues are marked with an asterisk (*). The cysteine residues are highlighted in black, catalytic residues (Asn 9, Lys 11, His96 and Glu 167) are highlighted in blue. A deletion of seven amino acids present in TPIs from *Synechocystis* and *Nostoc* is colored in red. Secondary structure is shown as follows: red cylinders for α-helix and green arrows for β-strands. **(B)** Size-exclusion chromatography of SyTPI (orange trace) in comparison to dimeric AtcTPI (gray trace), SyTPI and AtcTPI elute at 9.9 and 10.1 mL, respectively as a dimer, SyTPI and AtcTPI peaks were separated in a Superdex 75 10/300 column. The elution profiles for MW standards: conalbumin (75 kD), ovalbumin (43 kDa), carbonic anhydrase (29 kDa), and Ribonuclease A (13.7 kDa) are represented as dots. **(C)** Modulation of SyTPI enzymatic activity after treatment for 4 h at 25°C with four redox agents, 1 mM diamide (DA) (dotted bar), 25 mM oxidized glutathione (GSSG) (gray bar), 0.5 mM H_2_O_2_ (dark gray bar), and 0.5 mM methyl methanethiosulfonate (MMTS) (white bar). **(D)** Differential effect of redox agent of SyTPI (black), AtcTPI (red), and AtpdTPI (blue) enzymatic activity after 4 h of incubation at 25°C with increasing concentration of (1) GSSG from 0 to 25 mM. (2) DA, from 0 to 1,000 μM, (3) MMTS, from 0 to 50 μM. The inset shows the inhibition of AtcTPI from 0 to 0.25 μM, and (4) H_2_O_2_ from 0 to 500 μM. The results are expressed as a percentage of the activity with respect to the untreated protein sample. Each experiment was performed by triplicate.

A multiple sequence amino acid alignment between SyTPI and TPIs from photosynthetic and model organisms illustrates that the amino acid conservation between these enzymes is centered around amino acids that correspond to the active site and loop 6 (also called phosphate binding loop; Figure 1). This loop suffers a hinge motion that assembles the TPI active site driving catalysis upon substrate binding (Lolis et al., 1990; Wierenga et al., 1992). This sequence alignment revealed that cyanobacterial TPIs present a deletion of seven amino acids (residues N195 to A201 in AtcTPI) in the loop that connects α-helix 6 with β-strand 7 (Figure 1A). This alignment shows that SyTPI harbors tree cysteines at positions 42, 127, and 176. The localization of these cysteines contrasts with the character of residues C13 and C218 in AtcTPI and C15 in AtpdTPI that are targets of glutathione *in vitro* and are not conserved with cyanobacterial TPIs (Lopez-Castillo et al., 2016; Figure 1A and Table S3).

In order to understand the kinetic and biophysical features of SyTPI, we recombinantly expressed this protein in an *E. coli* strain containing a deletion of its *tpi* gene (Sullivan et al., 2011). After two chromatographic steps, recombinant SyTPIs were purified to homogeneity with a yield of approximately 4 mg per liter of cell culture with a purity >95% (Figure S1). After removal of the affinity tag, recombinant SyTPI contains three extra amino acids (Gly, Pro, His) before its initial methionine.

The elution profile of recombinant SyTPI in a gel filtration column indicates that this protein assembles as a dimer with an apparent molecular mass of 54 kDa (Figure 1B). Thus, SyTPI as the majority of TPIs, being the exception TPIs from archaea that assemble as tetramers, assemble as dimers (Walden et al., 2001). In contrast to bacterial and eukaryotic TPIs, the catalytic constants of TPIs from cyanobacteria are scarce. Our steady-state kinetic measurements indicate that SyTPI catalyzes the G3P to DHAP interconversion (forward reaction) with a K_M_ of 0.8 mM and a turnover number (k_cat_) of 1,848 s^−1^. These catalytic constants correspond to a catalytic efficiency of 2.31 × 10^6^ M^−1^ s^−1^. The catalytic efficiency of SyTPI is similar to the catalytic efficiencies of TPIs from photosynthetic organisms like *C. reinhardtii* and cytosolic and plastidic TPIs from *Arabidopsis* (Zaffagnini et al., 2014; Lopez-Castillo et al., 2016). Thus, SyTPIs as all TPIs studied to date, are nearly perfect enzymes that interconvert substrate with diffusion as a rate-limiting step (Blacklow et al., 1988; Wierenga et al., 2010; Table 1).

**Table 1:** Catalytic parameters for G3P and DHAP interconversion by wild-type and mutant SyTPIs.

**Enzyme**	**Km(mM)**	**Kcat(s^−1^)**	**Kcat/Km(M^−1^ s^−1^)**
SyTPI	0.80 ± 0.02	1848 ± 43	2.31 × 10^6^
C42A	1.01 ± 0.03	1776 ± 42	1.76 × 10^6^
C127A	1.04 ± 0.08	1774 ± 56	1.71 × 10^6^
C176A	1.01 ± 0.05	1811 ± 17	1.79 × 10^6^
C42A/C127A	1.10 ± 0.03	1631 ± 53	1.48 × 10^6^
C42A/C176S	1.46 ± 0.02	2126 ± 62	1.46 × 10^6^
C127A/C176S	1.19 ± 0.08	683 ± 6	5.73 × 10^5^
C42A/C127A/C176S	1.40 ± 0.15	21.32 ± 1	1.52 × 10^4^

The enzymatic activities of TPIs from *Arabidopsis* and *C. reinhardtii* display a contrasting fate upon the exposure to oxidizing agents. For instance, AtcTPI is readily susceptible to oxidation by diamide (DA) and H_2_O_2_, whereas AtpdTPI and CrTPI are resistant to those oxidative agents (Zaffagnini et al., 2014; Lopez-Castillo et al., 2016). Incubation of AtcTPI with 1 mM of DA reduced its activity to < 5% of the untreated enzyme, whereas the addition of 1 mM DA has no effect on the activity of AtpdTPI (Lopez-Castillo et al., 2016). Oxidation of cysteines by H_2_O_2_ and DA are proposed to promote the formation to sulfinic or sulfonic acids (Kosower and Kosower, 1995; Poole, 2015). Based on the differences in susceptibility to oxidative agents among various TIMs, we measured the effect of oxidative agents on the enzymatic activity of SyTPI. We found that the catalytic activity of SyTPI is not affected by the addition of 1 mM DA or 0.5 mM H_2_O_2_, whereas its enzymatic activity decreases to 50% of the untreated enzyme upon treatment with 25 mM oxidized glutathione (GSSG) and 0.5 mM S-methyl methane thiosulfonate (MMTS) (Figure 1C). In order to compare the fate of SyTPI and plant TPIs in the presence of thiol-conjugating agents, we performed a kinetic study using different concentrations of oxidative agents. We found that SyTPI is less susceptible to the thiol-conjugating agents GSSG, MMTS, and diamide, than AtcTPI and AtpdTPI (Figure 1D). The enzymatic activity of SyTPI (at a protein concentration of 0.184 nM) decreases to 50% after a treatment with 25 mM GSSG, this contrast with the enzymatic activities of AtcTPI and AtpdTPI, that are entirely inhibited at the same GSSG concentration. MMTS has no inhibitory effect on the enzymatic activity of SyTPI at concentrations lower than 50 μM. In contrast, AtcTPI and AtpdTPI exhibited 50% of their original enzymatic activity at 0.005 and 18 μM of MMTS, respectively. This indicates that a 27-fold molar excess of MMTS inhibits 50% of the enzymatic activity of AtcTPI, but a much higher (~272,000-fold) molar excess of MMTS has no effect on the catalytic activity of SyTPI. Likewise, we found that DA at concentrations up to 1,000 μM do not decrease the enzymatic activity of SyTPI. Conversely, the enzymatic activities of AtcTPI and AtpdTPI decreased to 20 and 80% of the untreated enzymes at DA concentrations of 1,000 μM. The enzymatic activity of SyTPI is also not affected by incubation with H_2_O_2_. In contrast, the catalytic function of AtpdTPI is reduced by 20% upon treatment with 500 μM H_2_O_2_, while the activity of AtcTPI decreases by 20% in the presence of 180 μM H_2_O_2_ (Figure 1D).

In order to further determine if the activity of SyTPI is modulated by the conjugation of redox agents in any of its cysteines, we performed a time-course experiment using wild-type SyTPI in comparison to AtpdTPI and AtcTPI. SyTPI and AtpdTPI present no inhibition after 60 min of incubation time with GSSG, whereas AtcTPI is inhibited to 50% of the unconjugated enzyme values after 30 min. The same trend is observed with MMTS, where AtcTPI presents 50% of its untreated activity after an incubation time of 15 min. In the case of DTNB all TPIs are inhibited with similar rates (Figure S2). The differential in inactivation between the three thiol-conjugating agents indicates that AtcTPI contains accessible cysteines that have a proper chemical environment to be conjugated with GSSG and MMTS, whereas AtpdTPI and SyTPI contain accessible cysteines, but their chemical environment is not poised to react with GSSG or MMTS. Complete DTNB inhibition and a minimal decrease in activity upon GSSG is also observed in CrTPI (Zaffagnini et al., 2014). DTNB is a negatively charged reagent, MMTS is neutral, and GSSG drives intramolecular disulfide bond formation preferentially with thiolates than thiols (Poole, 2015).

### Crystal structure of SyTPI in complex with a substrate analog

**Figure 2:**
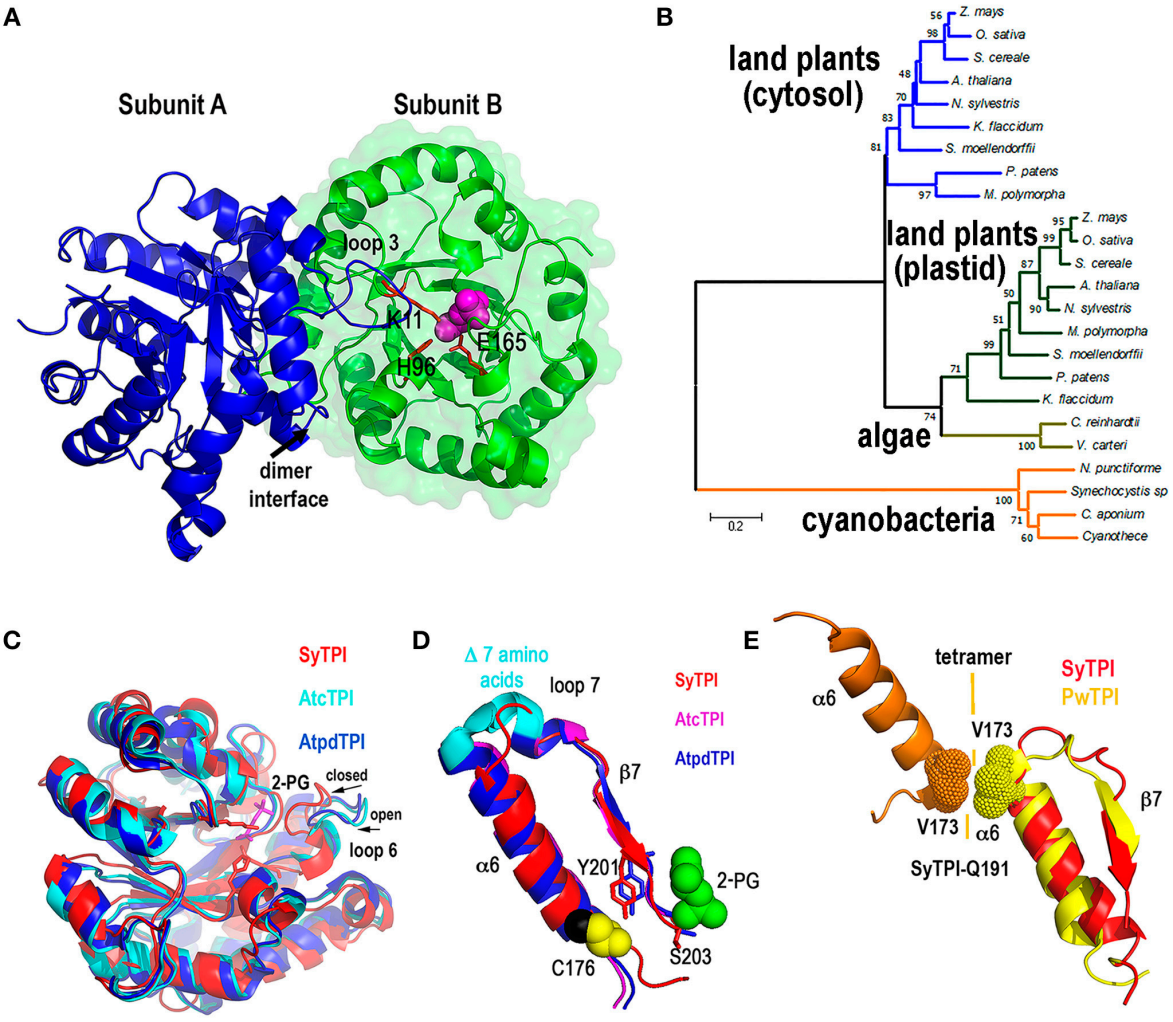
Quaternary structure of SyTPI. **(A)** Cartoon representation of homodimeric SyTPI. The dimer interface is formed by the interconnection of loop 3, subunit A is colored in blue and subunit B is colored in green. 2-PG is colored pink in space-filled representation and the catalytic residues Lys11, His 96, and Glu165 are represented in a ball-stick representation. **(B)** Phylogenetic tree of TPIs from photosynthetic organisms. The tree consists of representative amino acid sequences of TPIs from land plants, green algae, and cyanobacteria, highlighting those TPIs with known crystal structure [*A. thaliana* (cytosolic and plastidic), *C. reinhardtii, Synechocystis*]. Bootstrap values are reported at nodes. The distance scale is indicated between 0 and 1, where 0.2 indicates 20% differences between two sequences. **(C)** Superimposition of SyTPIs with AtcTPI and AtpdTPI. SyTPI is present in a closed loop 6 conformation. **(D)** Ribbon representation focus on α6 and β7 of SyTPI, AtcTPI, and AtpdTPI. AtcTPI and AtpdTPI connect α6 and β7 by means of a short α helix, whereas SyTPI presents a deletion of seven amino acids and connect these structural elements by a short loop. **(E)** Structural comparison between tetrameric TPI from *Pyrococcus woesei* (PDB: 1HG3) and SyTPI in the region that comprises α6 and β7. In PwTPI residue V173 of α6 is involved in assembling a tetramer. SyTPI harbors a glutamine (Q191) at the corresponding position.

In order to have a structural understanding for the insensibility of SyTPI to H_2_O_2_ and thiol-conjugating agents, we growth protein crystals of SyTPI in complex with 2-phosphoglycolate (2-PG) that diffracted at a resolution of 1.6 Å (Figure S1, Table S2). Resolution of the crystallographic data revealed that SyTPI assembles as a homodimer and that each monomer folds into a canonical (β-α)_8_ or TIM-barrel composed of eight α-helices and eight parallel β-strands (Figure 2A). Within the TIM barrel, the loops immediately after each β-strand assemble the front part of the barrel, and several of those loops are involved in dimer assembly (front loops 1, 2, 3, and 4) and catalytic activity (front loops 6, 7, and 8) (Katebi and Jernigan, 2014). The loops following each α-helix assemble the back part of the barrel and they play a structural role. The active site of SyTPI shows that the catalytic residues Lys 11, His96, and Glu165 are poised in close contact with the inhibitor 2-PG (Figure 2A). The total surface area of the dimer interface is 10,390 Å^2^. To have an evolutionary perspective of photosynthetic TPIs, we constructed a phylogenetic tree using TPIs from land plants, algae, and cyanobacteria (Table S4). The topology of the tree illustrates that cyanobacterial TPIs are not phylogenetically related with TPIs from plants and algae, as previously shown by Reyes-Prieto and coworkers (Reyes-Prieto and Bhattacharya, 2007). The topology of this tree also suggests that TPIs from green algae, like *C. reinhardtii*, have followed a distinct evolutionary trajectory in comparison to plastidic TPI form land plants (Figure 2B). A superimposition of the crystal structure of SyTPI with TPIs crystalized in the absence of ligand (AtcTPI and AtpdTPI) (Figure 2C) illustrates that SyTPI exhibits a closed loop 6 conformation (Mande et al., 1994; Lopez-Zavala et al., 2016). This superposition also indicates that the secondary structural elements of SyTPI are virtually identical between photosynthetic TPIs. As mentioned before, SyTPI harbors a deletion of 7 amino acids in the loop that connects α-helix 6 with β-strand 7. In AtcTPI and AtpdTPI this region folds as a short α-helix, whereas this region in SyTPI folds as a loop that connects α-helix 6 and β-strand 7 (Figure 2D). The absence of the short α-helix that connects α-helix 6 and β-strand 7 is shared with TPIs from archaea like *Pyrococcus woesei* (Walden et al., 2001). In TPIs from archea a conserved valine in α-helix 6 (V173 in *P. woesei*) is part of an interface that coordinately with residues from α-helices 4 and 5 assemble the tetrameric oligomer (Figure 2E). Although SyTPI lacks the short α-helix that connects α-helix 6 and β-strand 7, the residues and structural changes necessary for tetrameric assembly are not present in this protein. The structural localization in the back of the catalytic face of SyTPI of the connection between α-helix 6 and β-strand 7 suggest that a deletion of this α-helix should not have a role in catalysis.

### Structural accessibility, and reactivity of SyTPI cysteines

**Figure 3:**
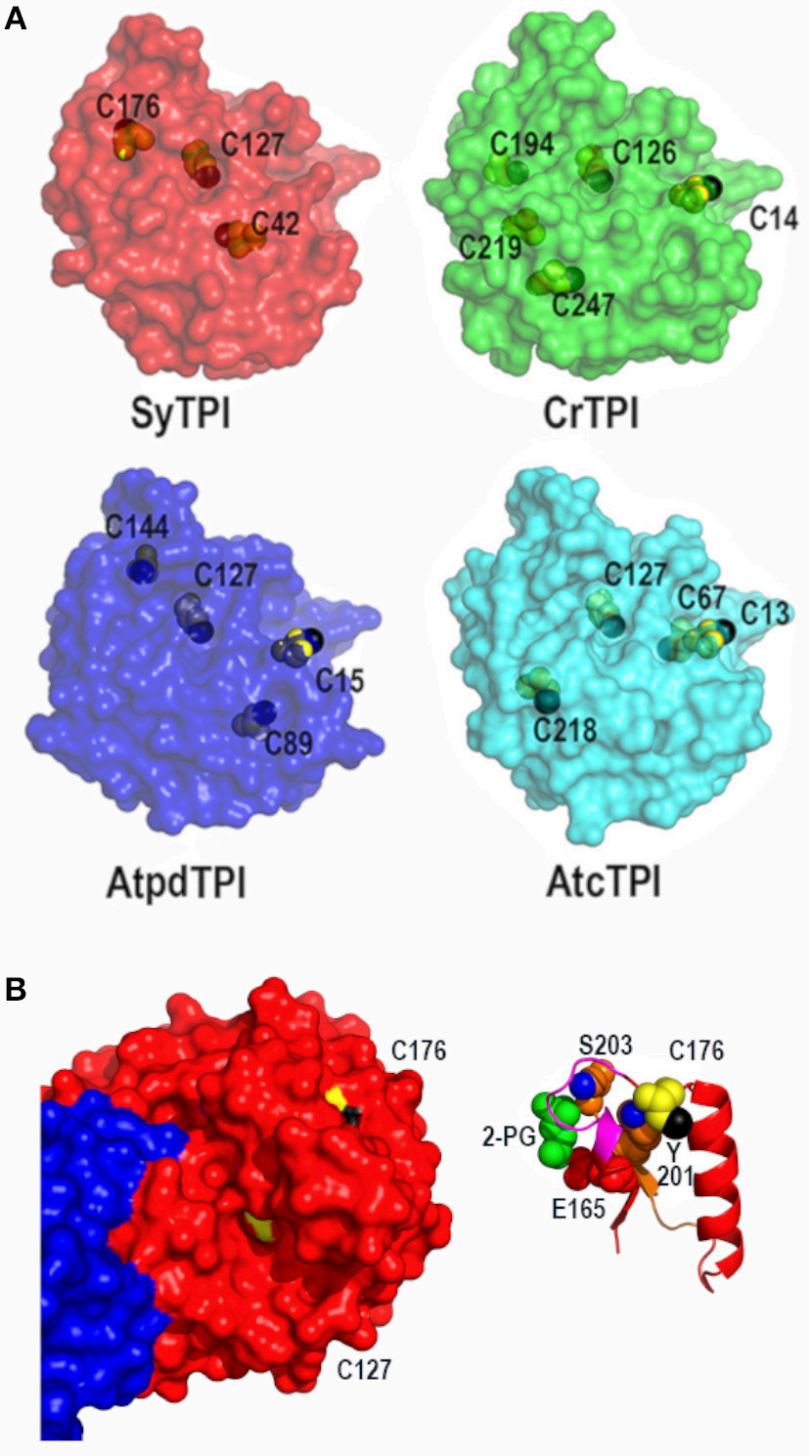
Crystal structure of dimeric SyTPI showing their cysteines. **(A)** Structural localization of SyTPI cysteines in comparison to the localization of cysteines from photosynthetic organisms (SyTPI, *Synechocystis* sp. PCC 6803 TPI; CrTPI, *C. reinhardtii* TPI; AtcTPI *A. thaliana* cytosolic TPI; AtpdTPI *A. thaliana* chloroplastic TPI). **(B)** Structural localization of SyTPI-C176. **(Left)** Surface representation of Subunits A and B of SyTPI colored in blue and red. All cysteine residues are colored in yellow. The thiol group of C127 points toward the hydrophobic core (black colored), whereas the thiol group of C176 is solvent exposed (black colored). **(Right)** Ribbon representation centered around C176. The side chain of residue C176 interacts with residues Y201. The hydroxyl group of Y201 (blue colored) and main chain of residue S203 stablishes van der Waals interactions with C176. Residues Y201 and S203 assemble the phosphate binding loop (loop 6).

To explain the redox-regulation of TPIs, several groups have focused on cysteine residues that could function as regulatory elements (Zaffagnini et al., 2014; Dumont et al., 2016; Lopez-Castillo et al., 2016). SyTPI contains three cysteines per monomer (Figure 3A). All TPIs contain a conserved cysteine in the position corresponding to SyTPI-C127(Olivares-Illana et al., 2017). The thiol group of this cysteine is completely buried in photosynthetic TPIs (Figure 3A) and in all TPIs crystallized to date (Olivares-Illana et al., 2017).

The localization of the cysteines in SyTPI differs with other photosynthetic organisms, that share a conserved cysteine located in loop1 (CrTPI-C14, AtpdTPI-C15, and AtcTPI-C13) (Zaffagnini et al., 2014; Lopez-Castillo et al., 2016) and a cysteine with potential to be S-glutathionylated (AtcTPI-C218 and CrTPI-C219) (Zaffagnini et al., 2014; Dumont et al., 2016; Lopez-Castillo et al., 2016; Figure 3A). The character of residues SyTPI-C42 and SyTPI-C176 are not conserved with CrTPI, AtcTPIs, and AtpdTPI (Figure 3A). Residue SyTPI-C42 is inaccessible to solvent, whereas residues SyTPI-C127 and SyTPI-C176 present solvent accessible surface areas (SASAs) of 2.4 and 1.7 Å^2^, respectively (Table 2). As mentioned earlier, the thiol group of residue SyTPI-C127 is completely buried, whereas the thiol group of SyTPI-C176 contains the only accessible thiol group in this protein (Figure 3B). A ribbon representation centered at residue SyTPI-C176, shows that this amino acid directly interacts with residues Y201 and S203 from the conserved loop 6 (phosphate-binding loop). The conformational change present in this loop is indispensable for catalysis and mutations of the corresponding residues of SyTPI-201 and SyTPI-S203 in yeast TPI severely hamper catalysis (Zhai et al., 2015). Consequently, structural changes in SyTPI-C176, mediated by point mutations of chemical modifications, should have a profound effect on catalysis.

**Table 2:** Theoretical solvent accessible surface area (SASA2) of cysteine containing residues in SyTPI calculate by ANCHOR (Meireles et al., 2010).

**Residue**	**SASA (Å^2^) of the dimer subunit A**	**SASA (Å^2^) of the dimer subunit B**
C42	0	0
C127	2.4	2.5
C176	1.7	1.7

In order to corroborate the predicted accessibility of cysteine residues, we measured the number of accessible thiols using Ellman's reagent [5, 5′-dithio-bis-(2-nitrobenzoic acid)] (DTNB) for wild-type and point-mutants SyTPIs (Table 3). We chose to mutate SyTPI-C47 and SyTPI-C127 to alanine because the thiol group of these residues is buried. We mutated SyTPI-C176 to serine, because this residue is exposed to the solvent environment. Substitution of residue C127 to serine in orthologous TPIs destabilizes their dimeric nature even more than an alanine mutation (Gonzalez-Mondragon et al., 2004; Hernández-Santoyo et al., 2012). This may be related to the hydrophobic character of cysteine and its inability to establish a hydrogen bond with water (Nagano et al., 1999). DTNB analysis revealed that wild-type SyTPI contains 1.37 accessible thiols per monomer. Point-mutant SyTPI-C127A decreases the accessible thiols to one, SyTPI-C42A to 0.88, and SyTPI-C176S to 0.55. The point mutants that decreases the most the number of accessible thiols is SyTPI-C176S, indicating that this residue contains the main accessible thiol in SyTPI. On the other hand, mutations of residues SyTPI-C42 and SyTPI-C176 present a minimal decrease in the number of accessible thiols (Table 3). In sum, the accessibility of cysteine thiols by DTNB indicates that residue SyTPI-C176 harbors an accessible thiol and that both residues SyTPI-C42 and SyTPI-C127 harbor a partially accessible thiol group.

**Table 3:** Accessible thiols in wild-type and mutants SyTPI determined by DTNB.

**Enzyme version**	**Accessible thiols/monomer**
SyTPI	1.37 ± 0.08
C42A	0.88 ± 0.02
C127A	1.01 ± 0.07
C176S	0.55 ± 0.05

### Mutation of SyTPI's cysteines reduce activity and stability

**Figure 4:**
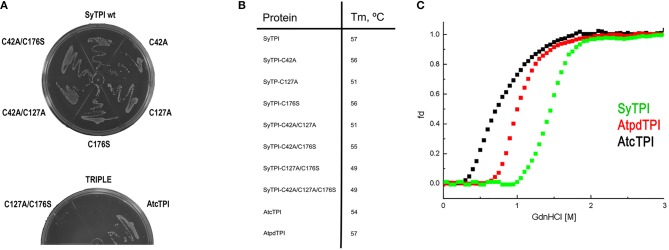
**(A)** LB agar plates showing the *in vivo* complementation of *E. coli* BL21 (DE3) Δtpi cells by plasmids expressing wild-type and mutants SyTPI and wild-type AtcTPI as positive control. **(B)** Thermal denaturation profiles of wild-type and point mutant SyTPIs in comparison to AtcTPI and AtpdTPI. **(C)** Gdn-HCl denaturation curves of SyTPI in comparison to AtcTPI and AtpdTPI. The y axes represent the percentage of unfolded protein (fd) and the x axis the Gdn-HCl concentration. Data measured the fluorescence of the samples at 280 nm.

In order to understand the effect of cysteine modification on SyTPI activity, we measured the catalytic activities of SyTPI mutants with decreased cysteine content (Table 1). We found that the catalytic activity single point mutants SyTPI-C42A, SyTPI-C127A, and SyTPI-C176S decreases 23–24% of that measured for the wild-type SyTPI. The enzymatic function of double mutants C42A/C127A and C42A/C176S is reduced by 1.56- and 1.58-fold, respectively, whereas the catalytic efficiency of the double mutant C42A/C176S and the triple mutant C42A/C127A/C176S decreases by 4.03- and 152-fold, respectively (Table 1). The kinetic parameters observed for SyTPI point mutants correlate with their ability to complement an *E. coli* Δ*tpi* strain (Sullivan et al., 2011). In this experiment, single point mutants of SyTPI are able of in *vivo* complementation, whereas no complementation is observed for the triple SyTPI C42A/C127A/C176S mutant and only a few colonies are present in the double C42A/C176S mutant (Figure 4A). None of the SyTPI cysteines is located near the active site. Thus, it is plausible that the observed decrease in activity for the double SyTPI-C42A/C176S and the cysteine-less SyTPI mutants is associated with a decrease in stability due to the accumulation of mutations (Bloom and Arnold, 2009). In order to investigate if the introduction of point mutants in SyTPI associates with a decrease in stability, we measured the stability or single, double and triple SyTPI mutants by a fluorescence-based thermal shift assay (Lo et al., 2004; Huynh and Partch, 2015). Wild-type SyTPI exhibited a melting point (Tm) of 57°C. Point mutations in residues SyTPI-C176S and SyTPI-C42, only reduce the Tm by 1°C, whereas single and double mutants containing the SyTPI-C127A substitution decrease their Tm by 6–8°C. The double mutant SyTPI-C127A/C176S and the triple mutant SyTPI-C42A/C127A/C176S present the most severe failures to complement *in vivo* and this correlates with a reduction in thermal stability (Figure 4B) and the reduced catalytic activity exhibited by those mutants (Table 1). Because a double point mutant renders SyTPI inactive, we wonder if SyTPI would present a decrease in stability with respect to TPIs from land plants. We determined their SyTPI, AtcTPI, and AtpdTPI stabilities by measuring the Gibbs free energy of folding upon chemical denaturation (Figure 4C). All TPIs displayed a two-state unfolding process, without the presence of appreciable intermediates. This observation suggests that quaternary interactions are necessary for stabilization of the folded monomeric state. In this case, a monophasic and protein concentration-dependent unfolding curves are expected. Therefore, denaturation profiles were analyzed using a two-state unfolding model (Table 4). This analysis indicates that the most stable protein is SyTPI, followed by AtpdTPI, while AtcTPI is the least stable protein. The Gibbs free energy of SyTPI implies that this protein is more resistant to unfolding by mutant accumulation with respect to AtcTPI or AtpdTPI.

**Table 4:** Thermodynamic parameters for unfolding data of SyTPI, AtcTPI, and AtpdTPI according to a two-state dimer denaturation model.

**Construct**	**Δ${\text{G}}_{D}^{H_{2}o}$ (kJ mol^−1^)**	***m* (kJ mol^−1^ M^−1^)**	^*^[GdnHCl] _50%_ (M)
SyTPI	64.8 ± 2.5	23.8 ±1.0	1.4 ± 0.1
AtpdTPI	50.5 ± 2.1	19.5 ± 1.5	1.0 ± 0.1
AtcTPI	41.2 ± 1.5	14.0 ± 1.0	0.72 ± 0.1

* *Concentration of GdnHCl necessary to get midpoint of denaturation transition*.

### Identification of accessible thiols by 2-nitro-5-thiocyanobenzoate (NTCB) footprinting

**Figure 5:**
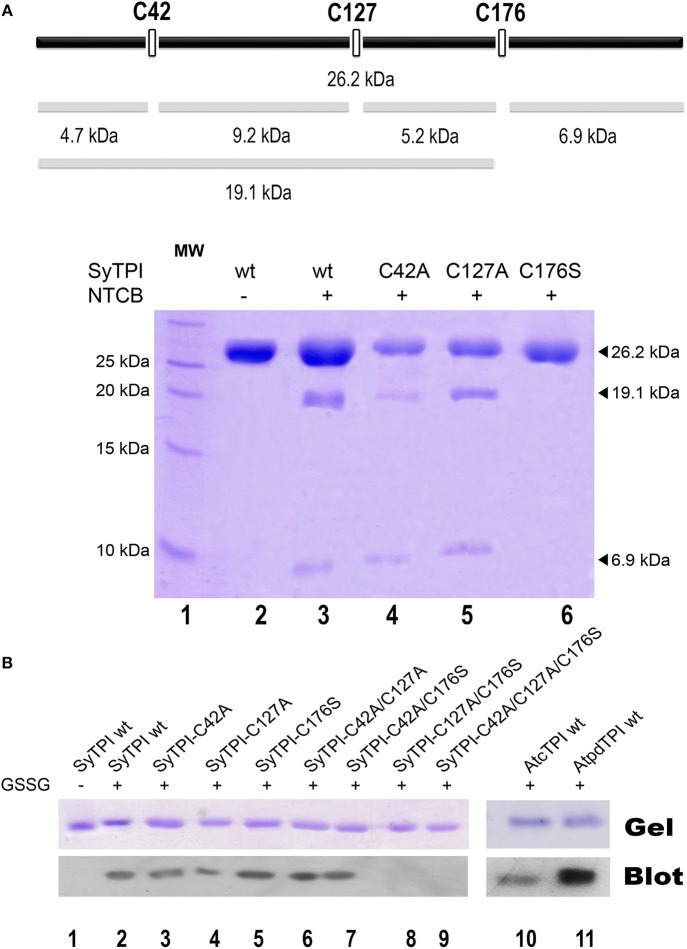
Biochemical determination of exposed cysteines in SyTPI. **(A)** NTCB cleavage fragments. The bars at the top provide a representation of the possible cleavage sites in SyTPI, samples were digested with NTCB and analyzed. 19.1 and 6.9 kDa NTCB cleavage fragments correspond to cleavage of residue cysteine 176 in wild-type SyTPI. **(B)** Identification of SyTPI glutathionylation by Western blot. Purified wild-type SyTPI and mutants were incubated in the presence of oxidized glutathione (GSSG). The proteins in the SDS-Page gel were transferred onto a nitrocellulose membrane for Western blot analysis, wild-type AtcTPI and AtpdTPI were used as positive control. Glutathionylation was detected for the wild-type SyTPI, single mutants and C42A/C127A and C42A/C176S double mutants (lanes 2–7) and positive control proteins (lanes 10–11), whereas no signal was detected for the double C127A/C176S and triple C42A/C127A/C176S mutant (lanes 8 and 9).

Our structural analysis indicates that SyTPI-C176 contains the only thiol group that is exposed to solvent. However, DNTB determination indicates that SyTPI contains more than one accessible cysteine per monomer (Table 3), so it is possible that SyTPI-C176 is a regulatory glutathione-binding site. To test this hypothesis, we determined the accessibility and reactivity of wild-type and single point mutants of SyTPI using 2-nitro-5-thiocyanobenzoate (NTCB) under native conditions. Cyanylation by NTCB of exposed cysteines following by the specific cleavage of the peptide bond at an S-cyanocysteine is used as a tool for protein footprinting (Tu and Wang, 1999). The expected molecular masses of the predicted cleavage proteolytic fragments assuming that all SyTPI cysteines are solvent exposed corresponds to fragments of 4.7, 9.2, 5.2, and 6.9 kDa (Figure 5A). We found that the NTCB footprinting reaction of SyTPI produced two proteolytic fragments of ~20 and 7 kDa. These fragments correspond to the theoretical molecular masses of proteolysis at residue C176, indicating that this residue contains the only thiol group that is solvent exposed. The footprinting reaction using single point mutants C42A and C127A presented an identical cleavage pattern than wild-type SyTPI, thus reinforcing the notion that NTCB only reacts with C176. This observation is further corroborated by the absence of proteolysis in the C176S mutant (Figure 5A).

Glutathionylation is a posttranslational modification that protects the oxidation of a thiol group and is a redox modification that can modulate enzymatic activity. TPIs from several species are targets of glutathione. In order to investigate if SyTPI is a target of glutathione, we tested if SyTPI and cysteine mutants could be glutathionylated. A biotinylated glutathione-specific antibody (BioGSSG) that interacts with glutathionylated proteins detected glutathionylated residues in SyTPI and the positive controls AtcTPI and AtpdTPI in a Western blot analysis (Figure 5B, lanes 1, 9, and 10). Glutathionylation was detected with the individual C42A, C127A, and C176S point mutants indicating that at least two cysteines could be conjugated with glutathionylation (Figure 5B, lanes 2, 3, 4). A signal was detected with the double mutants C42A/C127A, C42/176S, but not for the double mutant C127A/C176S and the triple mutant C42A/ C127A/C176S (Figure 5B, lanes 5, 6, 7, 8). This data indicates that both residues C176 and C127 are targets of glutathione *in vitro*.

### SyTPI, but not human TPI, is inhibited by ornidazole

**Figure 6:**
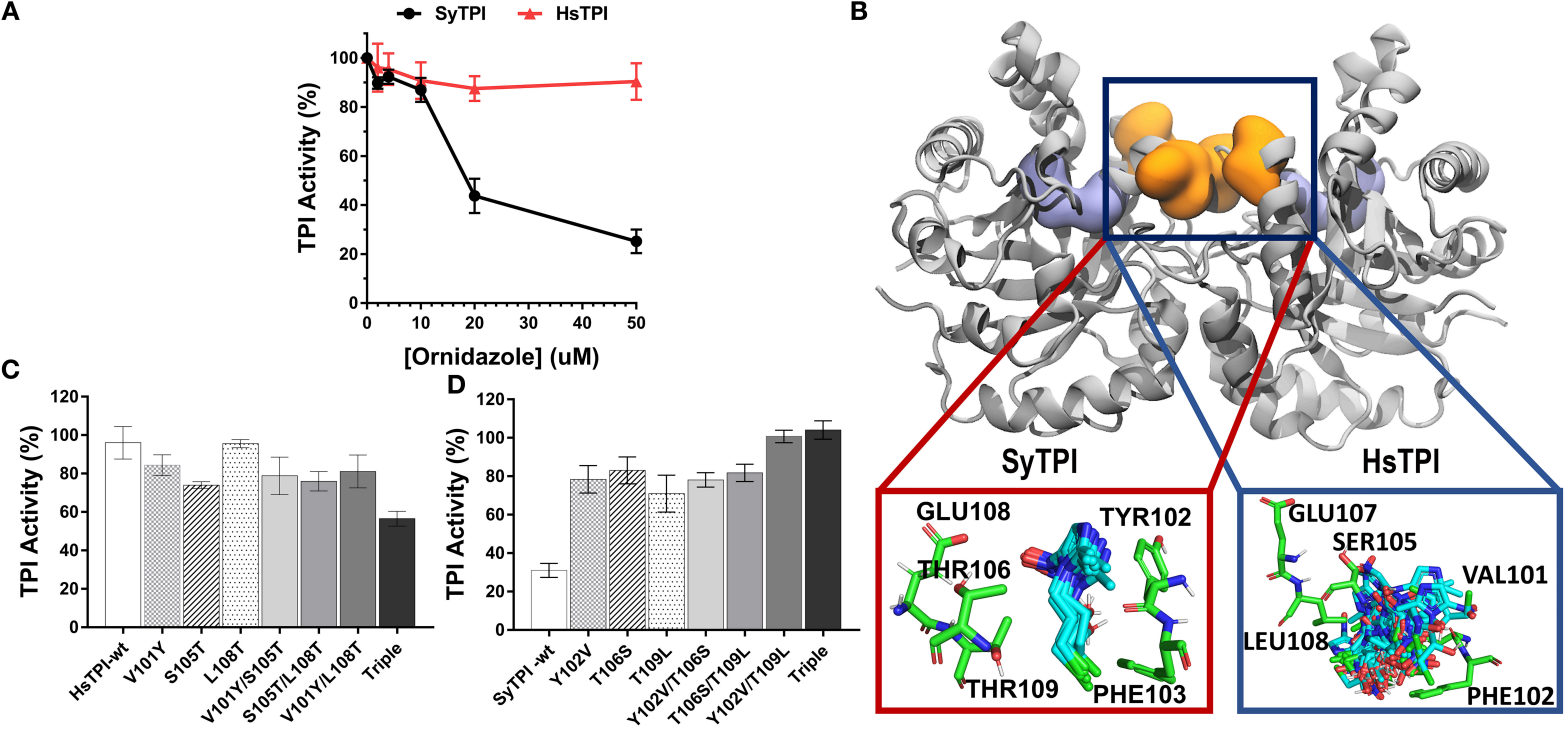
Structural mechanism for selective inhibition of SyTPI by ordinazole. **(A)** Residual TPI enzymatic activity of SyTPI (black) in comparison to HsTPI (red). TPI enzymatic activity was measured using increasing concentration of ornidazole from 0 to 50 μM followed by an incubation period of 60 min at 21°C. Results are expressed as mean percentage of the activity with respect to the untreated protein sample. Each experiment was performed by triplicate. **(B)** Localization of the ornidazole-binding site identified by ensemble docking simulations. The binding site for ornidazole is located at the interface of TPI in both SyTPI and HsTPI. In SyTPI, ornidazole adopts a consensus orientation in the interface and establish stable and specific contacts with the residues in this site (red box). Conversely, no specific ligand-protein contacts are found in the ornidazole-HsTPI complexes, leading to a highly heterogeneous spatial orientations of the inhibitor at the interface site of HsTPI (blue box). TPI is shown as gray ribbons and the ornidazole-binding site as orange surface. For reference, the location of the catalytic sites is shown as a violet surface representation. SyTPI and HsTPI residues as well as ornidazole are shown as sticks. **(C)** A few residues are responsible for the inhibitory effect of ornidazole in HsTPI. Relative triosephosphate activity for single, double and triple mutants that mimic the ornidazole binding site of SyTPI in HsTPI. **(D)** An inhibitory effect of ornidazole in SyTPI can be eliminated by site-directed mutagenesis. Relative triosephosphate activity for single, double and triple mutants that substitute the SyTPI ornidazole binding site by the corresponding residues present in HsTPI.

Small molecules can inactivate TPI in a non-competitive way by a mechanism that involves binding to the dimer's interface. Several studies have also shown that allosteric inhibition of TPI varies greatly among species; for example, benzothiazole derivatives at micromolar concentrations inhibit trypanosomal, but not human TPI (Téllez-Valencia et al., 2002, 2004; Alvarez et al., 2010). The selectivity of these small molecules is linked to the differences in the shape of the ligand-binding sites at the interface, which has led to the hypothesis that structural differences in TPIs from various species can be used to design selective small-molecule TPI inhibitors (Espinoza-Fonseca and Trujillo-Ferrara, 2006; Olivares-Illana et al., 2006). Recent work by Marcus and coworkers showed that ornidazole is a non-competitive inhibitor of SyTPI (Marcus et al., 2016), so we performed enzymatic assays to determine if ornidazole is a selective inhibitory of SyTPI. We compared residual triosephosphate isomerase of activity of human TPI and SyTPI using increasing ornidazole concentrations in the forward direction of the enzymatic reaction (e.g., G3P to DHAP, Figure 6A). We found that the enzymatic activity of SyTPI is sensitive to low micromolar concentrations of ornidazole, e.g., an ornidazole concentration of 50 μM reduces SyTPI activity by ~80% compared to the enzyme in the absence of inhibitor (Figure 6A). These findings are in agreement with a recent report showing that [ornidazole] = 25 μM decreases the V_max_ of SyTPI by nearly 80% (Marcus et al., 2016). In contrast, we found that ornidazole has a negligible effect on the enzymatic activity of human TPI (HsTPI) at inhibitor concentrations between 2.5 and 50 μM.

We used ensemble docking simulations to determine the structural mechanism for the ability of ornidazole to selectively inhibit SyTPI *in vitro*. We first used blind ensemble docking simulations to search for ornidazole-binding sites on both SyTPI and HsTPI. Analysis of the blind docking simulations revealed that ornidazole interacts with primarily two regions in both SyTPI and HsTPI: the catalytic site of the enzyme, and with a pocket formed between at the interface of the dimer (Figure 6B). However, it is unlikely that the catalytic site of the enzyme corresponds to the ornidazole-binding site because (a) this site is absolutely conserved in both SyTPI and HsTPI; (b) the TPI inhibition assays indicate that ornidazole does not inhibit HsTPI; and (c) previous studies have shown that ornidazole is a non-competitive inhibitor or SyTPI. This data and our blind ensemble docking simulations suggest that ornidazole likely binds at the interface of TPI. Therefore, we performed additional ensemble docking simulations focused on the interface of SyTPI and HsTPI. The focused ensemble dockings simulations revealed that ornidazole binds at the interface in a consensus orientation that is reproducible along the 100-ns trajectory of SyTPI. In this orientation, the -OH and -Cl moieties of ornidazole interact with Y102 and F103 of one SyTPI monomer, respectively; residues E108, and T106/T109 from the adjacent monomer interact with the -NO_2_ group and the aliphatic chain of ornidazole, respectively (Figure 6B). Ornidazole also interacts with HsTPI at the same site in the interface; however, we did not find specific interactions stabilizing the ornidazole-HsTPI complex, and ornidazole does not adopt a consensus orientation similar to that observed in SyTPI (Figure 6B). Analysis of the TPI structure generated through MD simulations revealed that the ornidazole-binding site at the interface is substantially more flexible in HsTPI than in SyTPI, which explains the inability of ornidazole to adopt a well-defined, consensus orientation on HsTPI. The formation of a well-defined ornidazole-binding site in SyTPI, and the absence of such site in HsTPI, correlate well with our experimental data showing that ornidazole selectively inactivates SyTPI. In order to test the hypothesis that SyTPI but not HsTPI, contained a defined ornidazole binding site, we constructed several HsTPI mutants that contain the SyTPI amino acids involved in ornidazole binding and SyTPI mutants that changes those amino acids to the ones present in HsTPI. We mutated residues Tyr102, Th106, Thr109 of SyTPI to valine, serine, and leucine, respectively to make the construct SyTPI-Hs-like and we mutated residues Val101, Ser105, and Leu108 of HsTPI to tyrosine, threonine, and threonine to make the construct HsTPI-Sy-like. We tested the inhibitory effect of ornidazole using individual, double, and triple mutants (Figures 6C,D). The triple mutant HsTPI-Sy-like decreases the percentage of activity from 90 to ~60%, suggesting that it is possible to recreate an ornidazole binding site by site-directed mutagenesis (Figure 6C). Conversely, the triple mutant SyTPI-Hs-like is not inhibited by the presence of 50 μM ornidazole (Figure 6D). During this analysis, single and double HsTPI mutants decrease their triosephosphate isomerase activity in the presence of ornidazole, whereas SyTPI mutants increase became not affected by the presence of ornidazole.

## Discussion

Redox-based signaling mechanisms, such as glutathionylation and nitrosylation, play an important role to regulate enzymatic activity under stress conditions. Redox-based signaling depends on the reactivity of exposed cysteine residues. In this study we found that the enzymatic activity of SyTPI is not affected by thiol-conjugating agents at concentrations where cytosolic and plastid TPIs are inhibited (Figure 1A). SyTPI is involved in the oxidative and reductive modes of carbon metabolism, meaning that is responsible for glycolysis and Calvin-Benson cycle, thus the integrity of this enzyme is critical in *Synechocystis*. Thus it is plausible to predict that cyanobacterial TPI evolved to avoid the irreversible oxidation of its cysteines to cysteine sulfinic or sulfonic acid (Zaffagnini et al., 2012b). Glutathionylation is a protective mechanism that prevents the irreversible oxidation of exposed cysteines, but glutathionylation is also a posttranslational modification that regulates enzymatic activity (Zaffagnini et al., 2012a,b, 2013; Rouhier et al., 2015; Moffett et al., 2017).

A comparison of the total and accessible cysteines in TPIs from photosynthetic organisms (Figures 1A, 3A) shows that the only conserved cysteine in photosynthetic organisms is the one that corresponds to SyTPI-C127. Our Western blot analysis indicates that residues SyTPI-C176 and SyTPI-C127 are glutathionylated *in vitro* (Figure 5B). This result contrasts with the NTCB cleavage pattern and the structural localization of the thiol group of SyTPI-C127 (Figure 5A). Glutathionylation of the residue analogous to SyTPI-C127 has been observed by proteomics studies in human, yeast, plant cytosolic, and cyanobacterial TPIs (McDonagh et al., 2009; Chardonnet et al., 2015; Dumont et al., 2016). Residue C127 is involved in dimer stabilization (Gonzalez-Mondragon et al., 2004; McDonagh et al., 2009; Samanta et al., 2011; Hernández-Santoyo et al., 2012). We hypothesize that glutathionylation of the conserved residue C127 in TPIs may be a product of transient exposure of its thiol group and is not a relevant target for glutathionylation.

**Figure 7:**
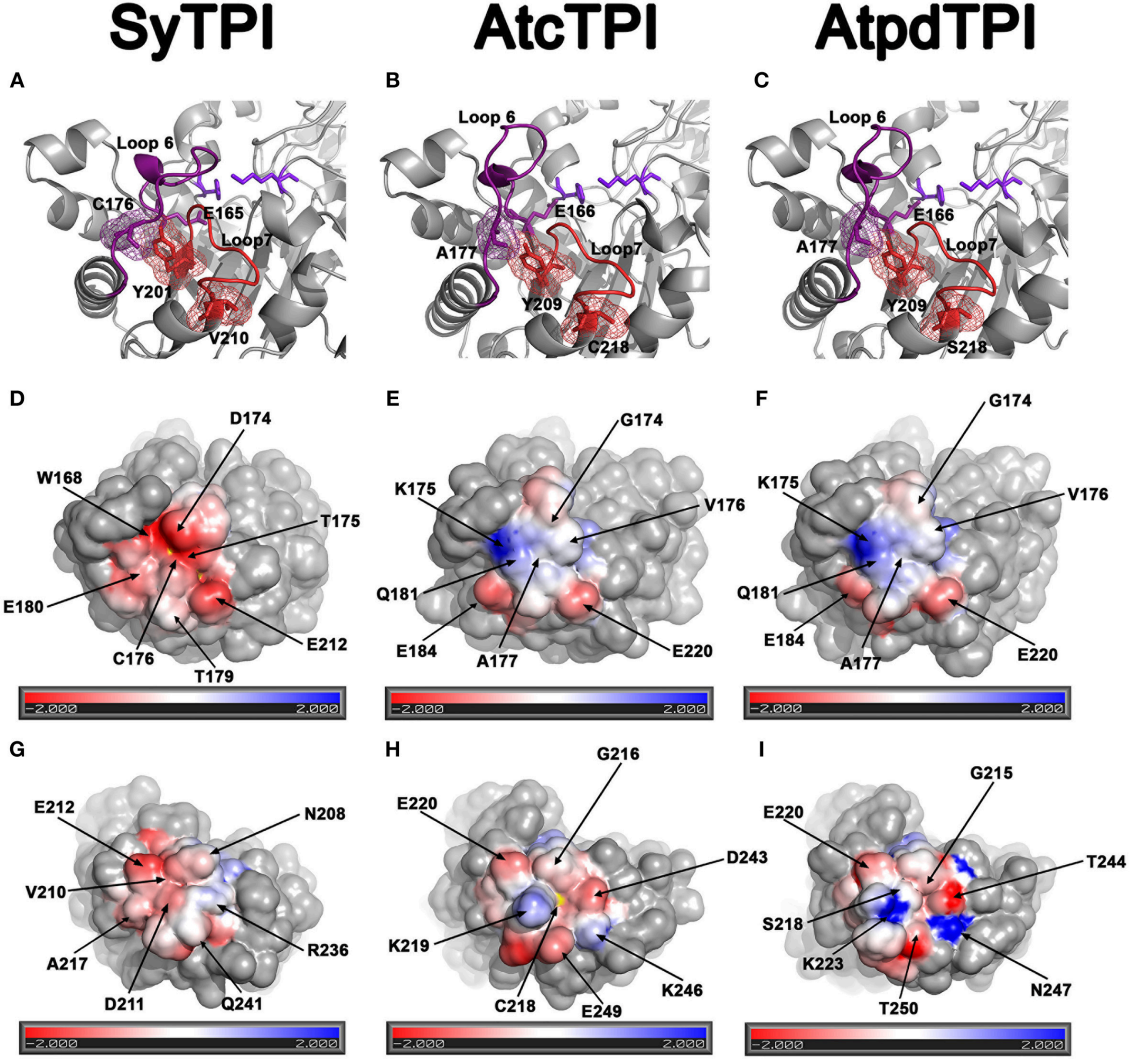
Structural basis for SyTPI resistance to redox agents. **(A–C)** Close up of the α-helix 7 showing residues SyTPI-V210 **(A)**, AtcTPI-C218 **(B)**, and AtpdTPI-S218 **(C)** colored in red in a stick representation, the three residues are located near a tyrosine (SyTPI-Y201, AtcTPI-Y209, and AtpdTPI-Y209) of the YGGS motif that makes contact with the respectively residues located immediately after (SyTPI-C176, AtcTPI-A177, and AtpdTPI-A177) to the catalytic residue (SyTPI-E165, AtcTPI-E166, and AtpdTPI-E166). Electrostatic potentials near residues SyTPI-C176, AtcTPI-A177, and AtpdTPI-A177 **(D–F)**, and SyTPI-A217, AtcTPI-C218, and AtpdTPI-S218 **(G–I)**. Negatively charged amino acids are colored in red and positively charged amino acid in blue. The thiol group of SyTPI-C176 and AtcTPI-C218 are colored in yellow.

AtcTPI, CrTPI, and AtpdTPI harbor a conserved cysteine (AtcTPI-C13, CrTPI-C14, and AtpdTPI-C15) located between β1 and α1 (Figures 7A–C). This cysteine presents different accessibility in related species like *T. cruzi* and *T. brucei* and it is a target for glutathionalyion in AtcTPI and AtpdTPI (Lopez-Castillo et al., 2016). AtcTPI also presents cysteine in position 218 (AtcTPI-C218) that is substituted by a serine in AtpdTPI. AtcTPI-C218 is postulated to be the main target for redox modifications in AtcTPI (Lopez-Castillo et al., 2016). The fact that SyTPI is not susceptible to redox agents, may be explained by the lack of a cysteine residue at a position equivalent to AtcTPI-C218. SyTPI presents its sole solvent exposed cysteine at residue, SyTPI-C176 (Figure 7A). SyTPI-C176 directly interacts with residues Y201 and S203 from loop 6, and the movement of this loop is indispensable for catalysis and mutations of the corresponding residues in yeast TPI hamper catalysis (Zhai et al., 2015). Thus, the main question is how SyTPI is not affected by modifications in residue SyTPI-C176. An electrostatic map near residue SyTPI-C176 (Figure 7D) shows that this residue is surrounded by amino acids that have a negative electrostatic potential (D174, E212, E180) limiting the potential of this residue to react with GSSG or with H_2_O_2_. In contrast, AtpdTPI and AtcTPI contain an alanine residue in the position corresponding to SyTPI-C176 (Figures 7D–F). Thus, SyTPI contains an accessible thiol, however its structural environment difficult its interaction with GSSG or H_2_O_2_. The inactivation of SyTPI by DTNB, but not GSSG and MMTS (Figure S1) may be related to the greater reactivity of DTNB. A similar phenomenon is observed in the conjugation of CrTPI by DTNB but not for GSSG, where incubation of CrTPI with DTNB abolishes enzymatic activity, but the treatment with GSSG does not affect the enzymatic activity of the enzyme. The notion that residue SyTPI-C176 is not a target of glutathione *in vivo* is corroborated by proteomic analysis (Chardonnet et al., 2015).

Glutathionylation of residue AtcTPI-C218 may modify the structural context of loop 7 that is in close proximity to the essential E166 (Lopez-Castillo et al., 2016). A close view of the amino acids near residue AtcTPI-C218 shows that this region mainly present and electronegative charge (Figure 7H). In SyTPI or AtpdTPI, this region does not contain a cysteine residue, but at alanine or a serine that could not be oxidized (Figure 7I).

On the basis of our structure-function analysis, we postulate SyTPI in plant chloroplasts was replaced by a duplicated copy of the cytosolic TPI because of its inability to be regulated by glutathionylation or redox-based compounds. Residue SyTPI-C176 is resistant to be modified by glutathionylation or redox agents because of its negatively charged structural environment. Thus, we speculate that the evolutionary pathway that nature took was to use the redox labile AtcTPI, but glutathione regulated, to evolve into a AtpdTPI resistant to redox agents but able to be regulated by glutathione possibly in residue AtpdTPI-C14. Additionally, we found that SyTPI is resistant to posttranslational modifications involving redox modifications of free cysteines, but not to the non-competitive inhibitor ornidazole (Marcus et al., 2016). Our structural modeling and side-directed mutagenesis studies suggest that SyTPI present an ornidazole binding site that is not present in HsTPI (Figure 6). We propose that ornidazole binds to a site at the interface of the dimer, in agreement with previous studies showing that several small-molecule allosteric TPI inhibitors act upon the enzyme's interface (Téllez-Valencia et al., 2004). Interestingly, inhibition of enzymatic activity does not occur in human TPI, which suggests that the lack of redox-sensitive sites in SyTPI might be compensated by the presence of allosteric sites that help modulate the activity of cyanobacterial TPIs.

## Author contributions

EC-T, ML-C, and NB-T execute experiments and interpreted data; PJ-S solve crystal structure, designed, and executed experiments; CB-C and ML-H execute experiments, wrote the manuscript, and interpreted data; EF-dG and LE-F performed the ensemble docking simulations and wrote the manuscript; AP-C performed mutagenesis and kinetic characterization; RS-M and AO-L conceived the project and wrote the manuscript; CD-Q protein purification; LB wrote the manuscript, conceived, and supervised the project.

### Conflict of interest statement

The authors declare that the research was conducted in the absence of any commercial or financial relationships that could be construed as a potential conflict of interest.

## Supplementary material

Figure S1 Purification of SyTPI by IMAC. Fifteen percent SDS-PAGE stained with Coomassie blue showing the IMAC purification step of SyTPI **(A)**. Crystals of SyTPI present a rod shape and reached maximal dimensions of 700 × 70 × 70 μm after 1 week **(B)**.

Figure S2 Time course reaction showing the effect of GSSG, MMTS, and DTNB treatment in the enzymatic activity of wild-type SyTPI, AtcTPI, and AtpdTPI.

Table S1 Q5 Site-directed mutagenesis oligonucleotides.

Table S2 Data collection and refinement statistics of Triosephosphate isomerase from Synechocystis in complex with 2-Phosphoglycolic acid.

Table S3 Access code for the amino acid sequences used to build the Multiple amino acid sequence alignment.

Table S4 Access code for the TPI sequences used to build the phylogenetic tree.
