# Supplementary figures and images for: Structural Basis for the Limited Response to Oxidative and Thiol-Conjugating Agents by Triosephosphate Isomerase From the Photosynthetic Bacteria Synechocystis
Source: Front Mol Biosci. 2018 Nov 27;5:103. doi: 10.3389/fmolb.2018.00103 (PMC6277545; doi:10.3389/fmolb.2018.00103)

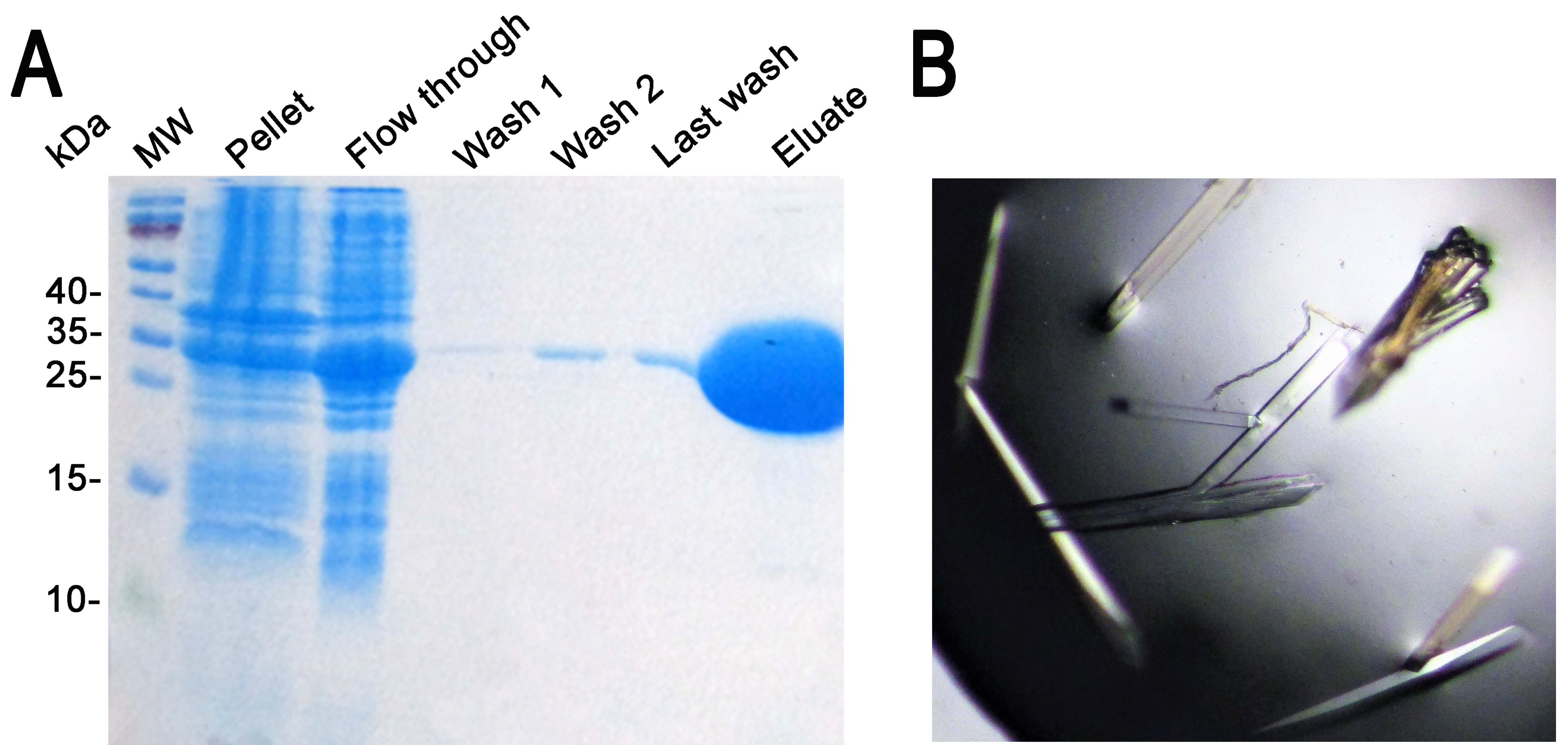

Supplement: Figure S1 — Purification of SyTPI by IMAC. Fifteen percent SDS-PAGE stained with Coomassie blue showing the IMAC purification step of SyTPI (A). Crystals of SyTPI present a rod shape and reached maximal dimensions of 700 × 70 × 70 μm after 1 week (B). [file Image_1.TIF]

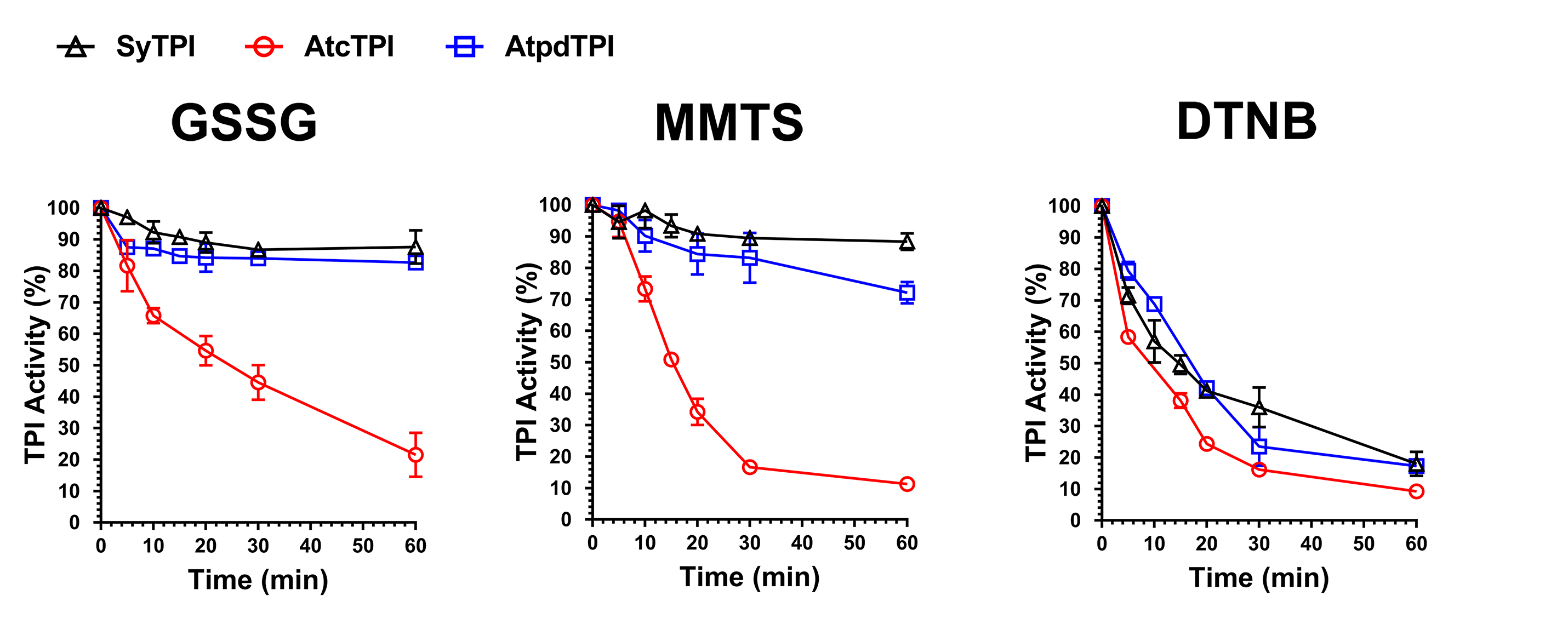

Supplement: Figure S2 — Time course reaction showing the effect of GSSG, MMTS, and DTNB treatment in the enzymatic activity of wild-type SyTPI, AtcTPI, and AtpdTPI. [file Image_2.TIF]
