# Supplementary material for: Structural Basis for the Limited Response to Oxidative and Thiol-Conjugating Agents by Triosephosphate Isomerase From the Photosynthetic Bacteria Synechocystis
Source: Front Mol Biosci. 2018 Nov 27;5:103. doi: 10.3389/fmolb.2018.00103 (PMC6277545; doi:10.3389/fmolb.2018.00103)
Supplement: Table S1 — Q5 Site-directed mutagenesis oligonucleotides. [file Table_1.docx]

| **Table S1\| Q5 Site-directed mutagenesis oligonucleotides.** | | |
| --- | --- | --- |
| SyTPI Tyr102Val | Up | ACGACGGCAAgttTTTGGGGAAACG |
|  | Lower | TCACTGTGGCCGATAACC |
| SyTPI Thr106Ser | Up | TTTTGGGGAAtcgGACGAAACTG |
|  | Lower | TATTGCCGTCGTTCACTG |
| SyTPI Thr109Leu | Up | AACGGACGAActtGCCAATTTGAGAGTATTG |
|  | Lower | TCCCCAAAATATTGCCGTC |
| SyTPI Thr106ser/Thr109Leu | Up | GGGGAAtCGGACGAActTGCCAATTTGA |
| SyTPI-Triple | \| Lower \| \| --- \| \| Up \| | AAAATATTGCCGTCGTTCACTGTGGCCG  ACGACGGCAAgttTTTGGGGAAtCGGACGAActTGCCAATTTGA |
|  | \| Lower \| \| --- \| \|  \| | TCACTGTGGCCGATAACCACATAATGAATACCGATTTCCGTC |
| HsTPI Val101Tyr  HsTPI Ser105Thr | Up  Lower  Up  Lower | GAGAAGGCATtacTTTGGGGAGTCAG  TCTGAGTGCCCCAGGACC  CTTTGGGGAGacaGATGAGCTGA  ACATGCCTTCTCTCTGAG |
| HsTPI Leu108Thr  HsTPI Ser105Thr/Leu108Thr  HsTPI-Triple | Up  Lower  Up  Lower  Up  Lower | GTCAGATGAGacgATTGGGCAGAAAG  TCCCCAAAGACATGCCTT  TTTGGGGAGacaGATGAGacgATTGGGCAGAAAG  GACATGCCTTCTCTCTGAGTGCCCCAGGACCACC  GAGAAGGCATtacTTTGGGGAGacaGATGAGacgATTGGGCAGAAAG  TCTGAGTGCCCCAGGACCACCCACGTGGCTCCGCAGTCTTTGATCATG |
|  |  |  |

| **Table S2\|** **Data collection and refinement statistics of Triosephosphate isomerase from *Synechocystis* in complex with 2-Phosphoglycolic acid.** | |
| --- | --- |
| **Identification** | **SyTPI** |
| **PDB code** | **6BVE** |
| **Wavelength** | 1.542 |
| **Resolution range** | 55.38 - 1.78 (1.844 - 1.78) |
| **Space group** | P 21 21 21 |
| **Unit cell** | 72.8799 73.0988 85.1753 90 90 90 |
| **Total reflections** | 358881 (27447) |
| **Unique reflections** | 44255 (4330) |
| **Multiplicity** | 8.1 (6.3) |
| **Completeness (%)** | 99.98 (99.98) |
| **Mean I/sigma(I)** | 16.93 (4.65) |
| **Wilson B-factor** | 15.54 |
| **R-merge** | 0.07286 (0.3655) |
| **Reflections used in refinement** | 44255 (4330) |
| **Reflections used for R-free** | 2006 (200) |
| **R-work** | 0.1543 (0.1888) |
| **R-free** | 0.1902 (0.2310) |
| **Number of non-hydrogen atoms** | 4091 |
| **Macromolecules** | 3723 |
| **Ligands** | 20 |
| **Solvent** | 348 |
| **Protein residues** | 484 |
| **RMS(bonds)** | 0.008 |
| **RMS(angles)** | 0.85 |
| **Ramachandran favored (%)** | 96.88 |
| **Ramachandran allowed (%)** | 2.71 |
| **Ramachandran outliers (%)** | 0.42 |
| **Rotamer outliers (%)** | 0.00 |
| **Clashscore** | 1.22 |
| **Average B-factor** | 17.26 |
| **Macromolecules** | 16.73 |
| **Ligands** | 17.29 |
| **Solvent** | 22.88 |

Statistics for the highest-resolution shell are shown in parentheses.

| **Table S3** Access coded for the amino acid sequences used to build the Multiple amino acid sequence alignment | | |
| --- | --- | --- |
| **Organism** | **Uniprot accession number** | **Alignment ID** |
| *Synechocystis sp* PCC 6803 | Q59994 | SyTPI |
| *Nostoc punctiforme* | B2JA20 | NoTPI |
| *Arabidopsis thaliana* cytosolic | P48491 | AtcTPI |
| *Arabidopsis thaliana* plastid | Q9SKP6 | AtpdTPI |
| *Chlamydomonas reinhardtii* | Q5S7Y5 | CrTPI |
| *Saccharomyces cerevisiae* | POO942 | ScTPI |
| *Trichomonas vaginalis* | A2EGX9 | TvTPI |
| *Homo sapiens* | P60174 | HsTPI |

**Table S4.** Access code for the TPI sequences used to build the phylogenetic tree

| **Organism** | **Identification code** |
| --- | --- |
| *Synechocystis sp* PCC 6803 | Q59994* |
| *Nostoc punctiforme* | B2JA20* |
| *Cyanobacterium aponinum* | K9Z135* |
| *Cyanothece sp* PCC 7822 | E0U7I7* |
| *Volvox carteri* | Vocar.0001s0851.1^+^ |
| *Chlamydomonas reinhardtii* | Q5S7Y5* |
| *Chlorella variabilis* cytosolic | E1ZKB3* |
| *Chlorela variabilis* plastid | E1ZJ95* |
| *Oryza sativa* cytosolic | P48494* |
| *Oryza sativa* plastid | LOC_Os09g36450.1^+^ |
| *Zea mays* cytosolic | P12863* |
| *Zea mays* plastid | PWZ12483.1* |
| *Secale cereale* cytosolic | P46226* |
| *Secale cereale* plastid | P46225* |
| *Nicotiana sylvestris* cytosolic | A0A1U7WBV5* |
| *Nicotiana sylvestris* plastid | A0A1U7XMB3* |
| *Arabidopsis thaliana* cytosolic | P48491* |
| *Arabidopsis thaliana* plastid | Q9SKP6* |
| *Sellaginella moellendorffii* cytosolic | XP_002960386- |
| *Sellaginella moellendorfii* plastid | D8SSS6* |
| *Marchantia polymorpha* cytosolic | Mapoly0042s0045^+^ |
| *Marchantia polymorpha* plastid | A0A176WT02* |
| *Physcomitrella patens* cytosolic | XP_024402314.1^-^ |
| *Physcomitrella patens* plastid | XP_024398301.1^-^ |
| *Klebsormidium flaccidum* cytosolic | GAQ81916.1^-^ |
| *Klebsormidium flaccidum* plastid | A0A1Y1HSX2* |

*Uniprot accession number
^+^Gene ID Phytozome 12
^-^NCBI accession number
